# Spatiotemporal modeling of regional short-term pertussis transmission risk using a propagation-enhanced prediction framework

**DOI:** 10.3389/fpubh.2026.1753050

**Published:** 2026-03-20

**Authors:** Siheng Zhang, Yao Zhu, Yan Xu, Yang Zhou, Hanqing He, Ying Xiao

**Affiliations:** 1 Zhejiang Provincial Center for Disease Control and Prevention, Hangzhou, Zhejiang, China; 2 Faculty of Medicine, Macau University of Science and Technology, Taipa, Macao SAR, China

**Keywords:** disease risk mapping, pertussis, public health surveillance, short-term forecasting, spatial propagation modeling, spatiotemporal prediction

## Abstract

**Introduction:**

Accurate forecasting of regional pertussis transmission is essential for public health surveillance, yet remains challenging because of complex spatial heterogeneity, implicit cross-city diffusion pathways, and nonlinear temporal dynamics.

**Methods:**

A spatiotemporal risk prediction framework was developed to model fine-grained disease spread across cities. The framework combines three-dimensional convolutional embeddings for local spatial pattern extraction, a Mamba-based state space architecture for temporal dependency modeling, and a propagation-aware spatial module constructed from administrative adjacency relations to characterize potential cross-city diffusion. In addition, a spatiotemporal mixed output head was introduced to enhance structural alignment and gradient continuity through state fusion and sequence permutation.

**Results:**

Experiments conducted on real pertussis heatmap sequences from Zhejiang Province showed that the proposed framework outperformed existing methods, achieving an MSE of 0.000930, an MAE of 0.020062, a PSNR of 31.057, and an SSIM of 0.9753. Ablation experiments further demonstrated that each module contributed consistently to performance improvement, while significance analysis confirmed the statistical reliability of the gains. Robustness evaluation under incomplete data conditions indicated that the model maintained strong predictive consistency even when the missing rate reached 15%.

**Discussion:**

The results demonstrate that the proposed framework can effectively capture spatial transmission structure and temporal evolution patterns of pertussis risk. Its strong accuracy, robustness, and stability indicate practical value for supporting regional infectious disease monitoring and public health decision-making.

## 1 Introduction

In recent years, regional-scale transmission forecasting of respiratory infectious diseases has become an essential component of public health surveillance, especially as highly contagious diseases such as pertussis show signs of localized resurgence (1, 2). Unlike many respiratory infections with relatively stable seasonal baselines, pertussis has re-emerged in multiple settings despite long-standing vaccination programs, where waning immunity, pathogen adaptation, and shifts in circulating strains can jointly reshape transmission intensity and spatial spread (3–5). These mechanisms often produce heterogeneous, city-level surges and spatially uneven diffusion, making pertussis particularly suitable for high-resolution spatiotemporal modeling that can track daily changes in risk surfaces. Accurately characterizing cross-city diffusion pathways and daily risk intensities is of great importance for resource allocation, intervention planning, and early warning. For example, heatmap-level daily predictions can support targeted deployment of diagnostic capacity and contact-tracing teams to emerging hotspots, and can also inform prioritized catch-up vaccination or intensified surveillance in high-risk cities identified by rising risk gradients (6–8). Traditional periodic statistical reporting or case-aggregation approaches often fail to capture fine-grained spatial structures across regions (6, 7, 9). In contrast, heatmap-based spatiotemporal forecasting methods provide a visual representation of the dynamic evolution of disease burden within a spatial layout, enabling sequence prediction models to learn city-level propagation patterns at a higher level of abstraction (10–13). This offers more timely and interpretable risk assessment support for local public health departments.

However, existing spatiotemporal forecasting models still face several limitations when applied to real-world public health data. On the one hand, many models rely heavily on local convolutions or short-range temporal modeling, making it difficult to capture both the slow cross-city diffusion and the rapid intra-city fluctuations that commonly coexist in pertussis transmission (14). On the other hand, most methods lack explicit modeling of administrative adjacency structures, preventing them from leveraging inherent geographic interactions between cities. As a result, the predicted heatmaps often suffer from discontinuous boundaries, weak directional cues, and insufficient spatial consistency. Importantly, these modeling limitations are amplified by practical constraints in surveillance systems, including delayed case reporting and heterogeneous reporting quality across jurisdictions, which can distort the observed spatiotemporal signals and weaken the reliability of short-term risk tracking in operational settings (15, 16). Moreover, challenges such as missing data, reporting delays, and highly heterogeneous spatiotemporal distributions further complicate model adaptation to realistic surveillance environments.

To address these issues, this study proposes a spatiotemporal prediction framework that integrates three-dimensional convolutional embeddings, state-space modeling, and propagation-aware enhancement mechanisms to construct a risk heatmap prediction model that better aligns with the characteristics of infectious disease diffusion. Specifically, we first employ a 3D-CNN to extract local spatial textures and short-term dynamic features, followed by a Mamba-based state-space module to efficiently capture temporally coherent state evolution patterns and robust spatiotemporal representations under non-stationary and noisy dynamics. A propagation-aware spatial module is then designed based on administrative adjacency relations to explicitly encode potential cross-city diffusion pathways. Finally, a spatiotemporal mixed-state output head is introduced to improve gradient continuity and structural alignment through state fusion and sequence permutation operations. The overall framework aims to achieve multi-scale collaborative modeling from local to global patterns and from structural relations to directional dynamics.

The main contributions of this work are summarized as follows:

(1) We propose a spatiotemporal forecasting framework tailored for public health scenarios, combining 3D-CNNs, state-space modeling, and explicit propagation-structure encoding to enable fine-grained prediction of pertussis risk heatmaps.
(2) We design a propagation-aware spatial module that leverages administrative adjacency topology to enhance the modeling of cross-city diffusion relationships, effectively improving directional coherence and spatial consistency in the predictions.
(3) We develop a spatiotemporal mixed-state output head that strengthens structural restoration and gradient reconstruction through sequence reordering and global state fusion.
(4) We construct a real pertussis heatmap dataset and conduct systematic evaluations, including comparative experiments, ablation studies, significance analysis, and missing-data robustness assessment, to validate the effectiveness and stability of the proposed approach from multiple perspectives.

## 2 Related work

### 2.1 State-space sequence models and spatial propagation modeling

In recent years, state-space sequence models (SSMs) have demonstrated remarkable advantages in sequence modeling. Their core idea lies in capturing temporal dependencies through linear recurrence and structured state-update mechanisms. Representative work, such as the Structured State Space Models proposed by Gu et al., enables efficient processing of sequences with low computational complexity, offering a new pathway for modeling complex dynamical processes (17). Subsequently, Mamba further introduced a selective state-space structure to achieve linear-time inference, significantly outperforming the Transformer attention paradigm in sequence tasks (18). Meanwhile, the SSM framework has been extended to domains such as reinforcement learning and continuous-time modeling, highlighting its generality and stability in high-dimensional dynamical systems (19, 20). Collectively, these studies suggest that state-space models possess inherent strengths in handling tasks characterized by large temporal spans and complex dynamic structures, providing a solid theoretical foundation for modeling of infectious disease transmission trends. We also acknowledge classical epidemiological approaches, including Bayesian hierarchical disease-mapping models and spatial autoregressive priors such as CAR/SAR, which provide principled spatial dependence modeling and uncertainty quantification at the areal level (21–23). However, these formulations are commonly built on region-level response variables and pre-specified dependence structures, and may be less suited for learning highly non-linear multi-scale diffusion patterns or directly reconstructing dense high-resolution spatial fields, motivating data-driven deep architectures for pixel-level heatmap forecasting.

In terms of spatial propagation modeling, extensive research has characterized infectious-disease spread from the perspectives of graph structures, mobility networks, and regional dependencies. Systematic surveys on spatiotemporal graph deep learning point out that graph structures effectively encode spatial correlations among regions, enabling models to better perceive propagation routes and diffusion patterns during prediction (24). Methodologically, the Spatial Propagation Network (SPN) proposed by Liu et al. demonstrates the potential of learnable spatial diffusion based on adjacency relationships (25). In practical applications, spatiotemporal graph neural networks (ST-GNNs) have been widely adopted for regional disease forecasting in contexts such as COVID-19 and influenza (14, 26, 27). These studies collectively highlight the crucial role of spatial adjacency and inter-regional dynamic interactions in modeling epidemic spread.

Furthermore, recent research continues to emphasize the importance of mobility data, dynamic graph structures, and phylogenetic information in forecasting transmission dynamics. Zhang et al. introduced dynamic virtual graph structures to improve contextual sensitivity in influenza spread modeling (28), Jiao et al. combined LSTM and attention mechanisms to achieve notable performance in mobility-driven spatiotemporal prediction (29). Overall, existing studies increasingly highlight the importance of spatial topology, diffusion pathways, and temporal dependencies in regional-level epidemic forecasting, yet a clear gap remains in extending spatial propagation dynamics to pixel-level heatmap prediction. Specifically, most existing epidemic forecasting models are designed to predict discrete node-/region-level scalar quantities on graphs or areal partitions (14, 26, 27, 30, 31), and therefore do not explicitly optimize the reconstruction of a dense *H*×*W* spatial field with fine-grained gradients, coherent boundaries, and temporally consistent diffusion fronts that are required for high-resolution risk-surface forecasting. Building upon these theoretical and methodological advances, this work proposes integrating state-space models with learnable spatial propagation mechanisms to establish a new modeling paradigm for fine-grained infectious-disease heatmap forecasting.

### 2.2 Spatiotemporal deep learning for epidemic forecasting

With the rapid development of deep learning in spatiotemporal forecasting, an increasing number of studies have attempted to leverage convolutional networks, recurrent architectures, and attention mechanisms to capture the complex spatiotemporal dependencies inherent in infectious disease transmission. Muñoz-Organero et al. employed deep spatiotemporal models to enhance regional COVID-19 forecasting performance (32), while Paul et al. utilized a ConvLSTM ensemble to model multivariate epidemic spread (33). Sciannameo et al. applied deep learning methods to predict the spatiotemporal evolution of new cases and hospitalizations in Italy, achieving strong results (34). Meanwhile, researchers have also explored the influence of external environmental factors on epidemic propagation—for example, Chen et al. integrated meteorological historical data into spatiotemporal forecasting models to improve high-resolution short-term predictions (35). At a broader geographical scale, Kavouras et al. modeled the spatiotemporal dynamics of COVID-19 across Europe using deep learning (36), and Ravenda et al. proposed a probabilistic spatiotemporal neural network that demonstrated stable performance in epidemic count forecasting (37). Together, these studies highlight the critical role of spatiotemporal deep learning in capturing epidemic transmission dynamics and demonstrate that sequence modeling combined with spatial convolution effectively represents both local temporal patterns and spatial neighborhood correlations during disease spread.

On the other hand, spatiotemporal modeling approaches that incorporate mobility data, graph structural information, and physical priors have received increasing attention. Jiao et al. constructed a behavior-sensitive epidemic forecasting framework by integrating mobility data, LSTM architectures, and attention mechanisms (29), while Nikparvar et al. employed deep LSTM networks for county-level COVID-19 forecasting in the United States (38). Furthermore, the introduction of graph neural networks has enhanced the modeling of inter-regional transmission relationships; for instance, Mao et al. proposed a backbone-based dynamic spatiotemporal GNN for epidemic prediction, illustrating the benefits of graph structures for multi-region spread modeling (39). Fujita and Akutsu incorporated physical information into spatial identity neural networks, improving the stability and interpretability of epidemic prediction (40). Overall, although existing methods can capture local spatiotemporal features or regional-level transmission pathways, they still lack a unified framework capable of integrating temporally coherent state evolution modeling, spatial structural understanding, and fine-grained spatial forecasting. Building on this foundation, this work further advances the field by constructing a spatiotemporal prediction system that integrates 3D-CNN spatial modeling with Mamba-based state-space sequence modeling for robust temporal integration under non-stationary dynamics, offering a new solution for high-precision disease heatmap forecasting.

## 3 Methods

### 3.1 Problem definition

In this study, we formulate the forecasting of regional pertussis heatmap sequences as a typical spatiotemporal prediction problem. Let {*X*_1_, *X*_2_, …, *X*_*t*_} denote the temporally ordered historical heatmaps of pertussis distribution, where each $X_{i}\in {\mathbb{R}}^{H\times W}$ represents the spatial grid–level case intensity on day *i*, reflecting the regional diffusion pattern of the disease. Here, the *H*×*W* grid is a purely geometric rasterization under a fixed coordinate system, where each cell corresponds to an equal-area spatial location in the map projection rather than uniform population exposure; population-weighted exposure adjustment is not explicitly imposed in this heatmap representation. Our objective is to learn a function *F*(·) that, given a historical observation window of length *T*, predicts the next heatmap as ${\hat{X}}_{t+1}=F\left(X_{t-T+1},\ldots ,X_{t}\right)$, generating high-quality and fine-grained spatial risk distributions while preserving spatial structure, propagation patterns, and temporal dependencies. The heatmap intensity in each cell encodes the city-level daily case count mapped to the corresponding polygon region and then normalized to a consistent scale to ensure comparability across dates, rather than a per-capita incidence measure. To this end, we propose a framework that integrates the local spatial and short-term dynamic modeling capability of 3D-CNN with the temporal state modeling mechanism of Mamba, and incorporates a spatial propagation path enhancement module at the output stage, enabling the model to capture both regional adjacency diffusion relationships and multi-scale temporal evolution trends for refined pertussis heatmap prediction.

### 3.2 Overall model architecture

**Figure 1:**
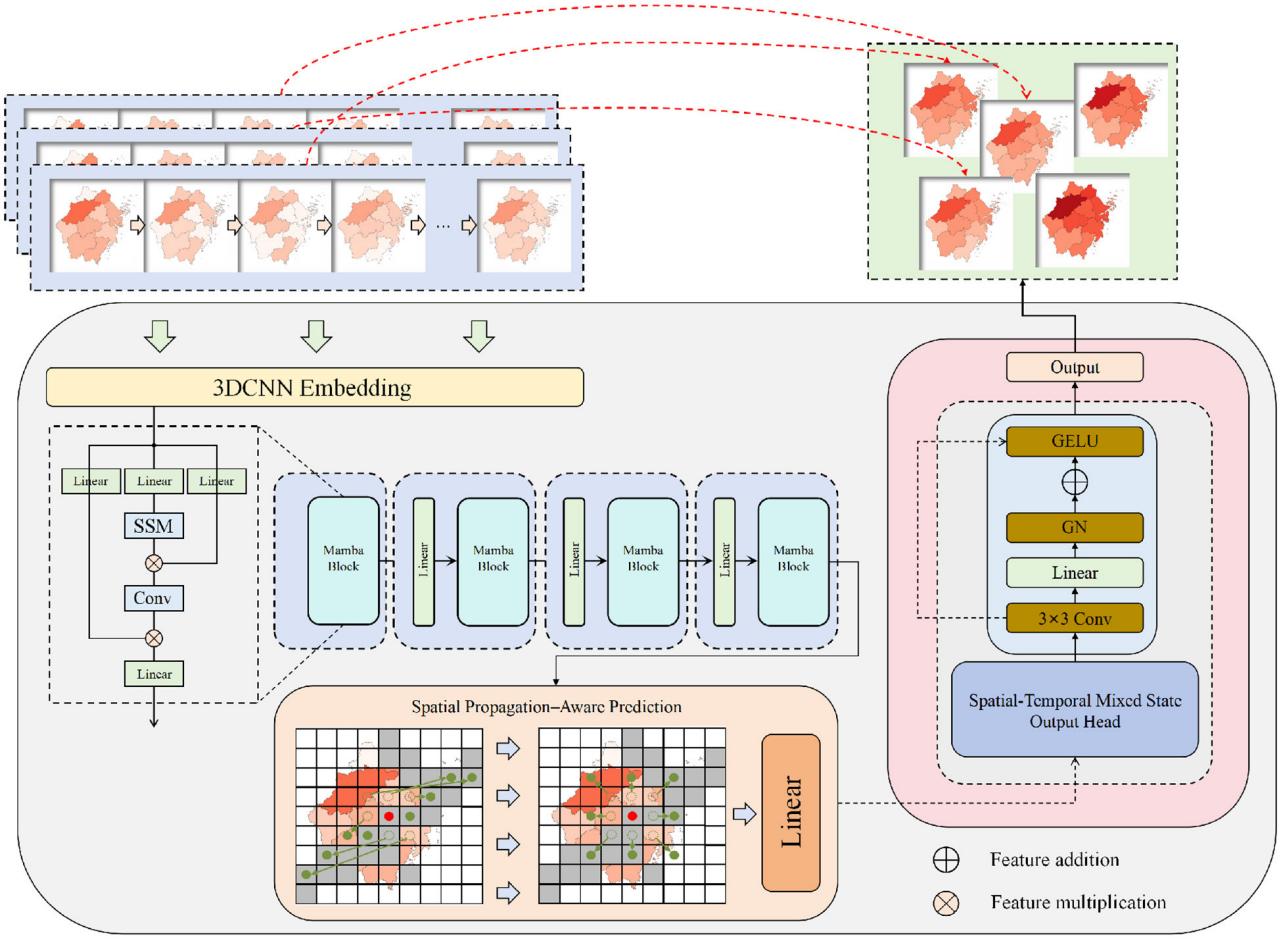
As shown in the figure, the proposed heatmap prediction framework for pertussis regions is illustrated. The model sequentially includes 3DCNN spatial-short-term dynamic embedding, Mamba state-space temporal modeling, a spatial propagation perception enhancement module, and a spatiotemporal hybrid output head. This system achieves multi-scale representation and propagation path modeling of historical spatiotemporal sequences through a unified end-to-end structure, thereby generating a refined risk distribution heatmap for the next time step.

**Figure 2:**
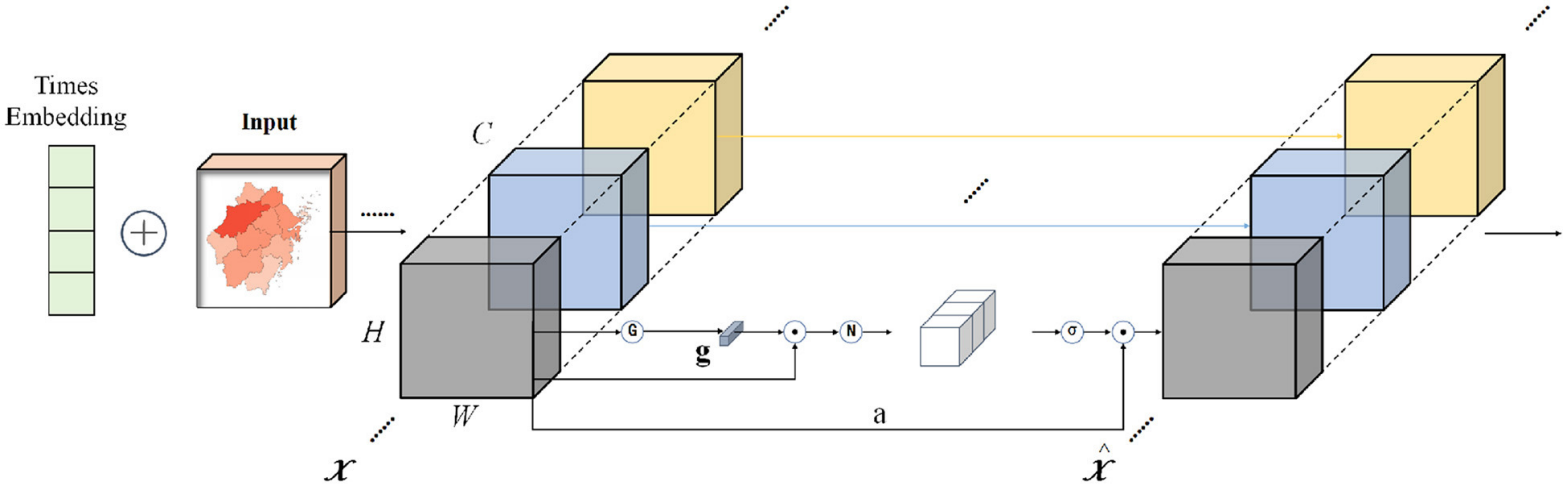
Example diagram of 3DCNN model architecture.

At the overall framework level, this study proposes an end-to-end deep network that maps historical pertussis heatmap sequences to the next-step regional risk distribution, as illustrated in Figure 1. Given an input sequence of length *T*, {*X*_*t*−*T*+1_, …, *X*_*t*_}, the model first extracts spatiotemporal feature representations that encode both spatial structure and short-term dynamics through a 3DCNN Embedding module. The architecture of the 3DCNN model is shown in Figure 2.

These representations are then passed into a Mamba-based state-space backbone to capture temporally coherent state evolution patterns and robust temporal integration under non-stationary dynamics, and multi-scale evolutionary patterns. After backbone processing, the resulting features flow into the Spatial Propagation–Aware Prediction module, which explicitly encodes regional adjacency relationships and potential diffusion pathways; simultaneously, they are combined with propagation-enhanced features and sent into the Spatial–Temporal Mixed State Output Head, which generates the predicted heatmap ${\hat{X}}_{t+1}$ through convolution, linear projection, and normalization-based activation. In this manner, the framework establishes a unified reasoning pipeline that covers spatiotemporal encoding, state evolution, and spatial propagation modeling.

The backbone of the model consists of a 3DCNN embedding stage followed by multiple stacked Mamba state-space blocks, jointly representing the spatiotemporal dynamics of pertussis transmission in the latent space. Let Φ_3D_(·) denote the 3D convolutional embedding operator, Proj(·) a linear projection along the channel dimension, and M(·) the sequence model composed of stacked Mamba Blocks. The backbone mapping of the input sequence can then be formalized as shown in Formula 1


$$H=M(\text{Proj}(\Phi_{\text{3D}}(X_{t-T+1},\ldots ,X_{t}))),$$ (1)

where *H* is a high-dimensional latent state tensor containing temporally coherent state evolution patterns and robust spatiotemporal representations. We adopt Mamba not merely for handling long contexts, but because its state-space recurrence and selective gating provide a strong inductive bias for non-stationary and noisy epidemiological dynamics, enabling stable information integration across time while preserving efficiency. In particular, the linear-time state update and input-dependent selection help suppress transient fluctuations and amplify persistent transmission signals, yielding a more reliable latent state for downstream spatial propagation enhancement and structured heatmap reconstruction. The subsequent spatial propagation–aware module and the spatiotemporal mixed output head operate on this latent state to produce a refined prediction of the next-step pertussis heatmap.

### 3.3 Spatial propagation–aware prediction

**Figure 3:**
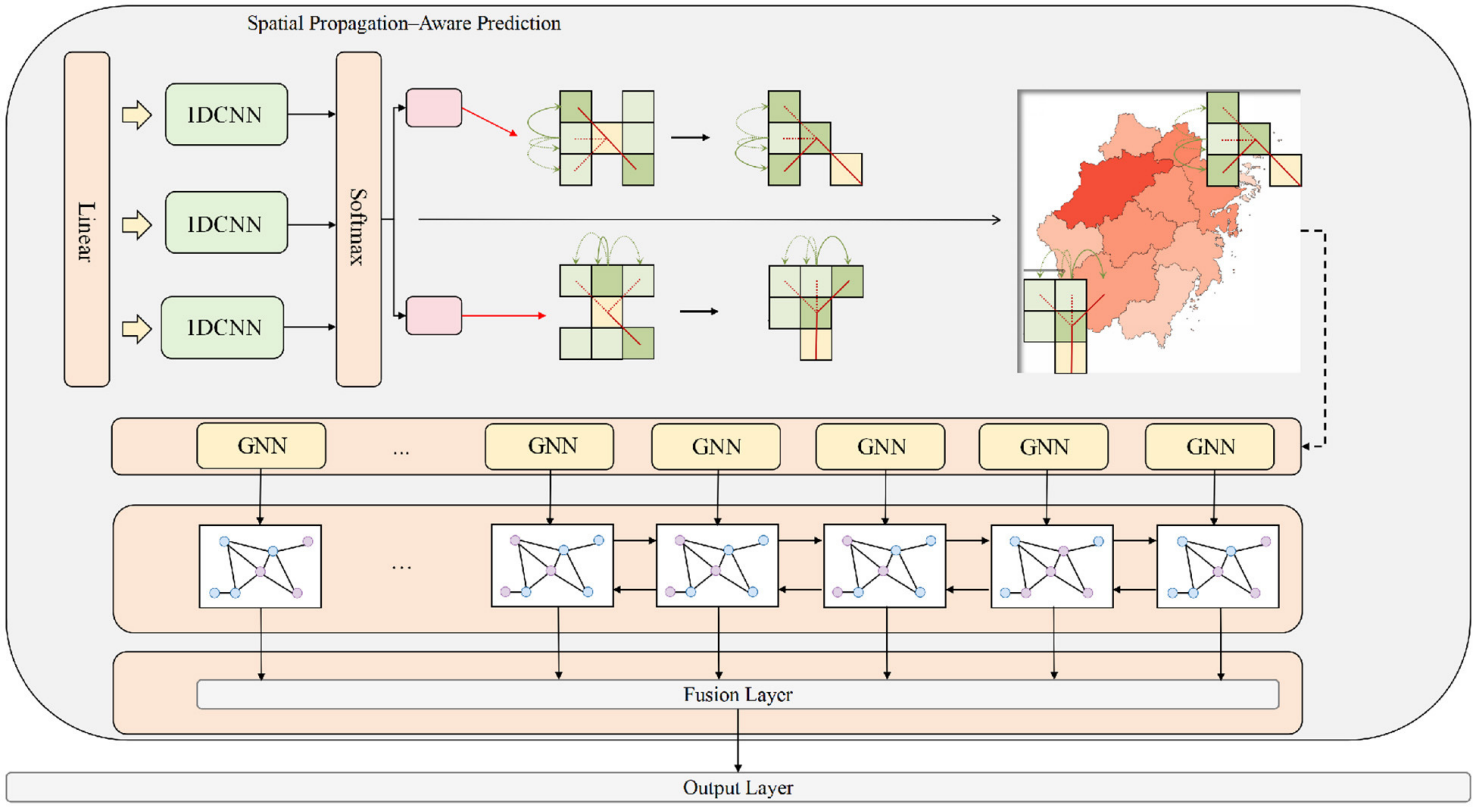
This figure illustrates the overall structure of the Spatial Propagation-Aware Prediction module, including multi-mode propagation weight extraction based on one-dimensional convolution, softmax-normalized generation of region propagation intensity, weighted construction of directional propagation templates, and spatial diffusion modeling of multi-layer graph neural networks. This module transforms the hidden states of the spatiotemporal backbone network into enhanced region-level representations by explicitly characterizing region adjacency relationships and potential propagation paths, thereby improving the spatial consistency and diffusion rationality of pertussis heatmap predictions.

After obtaining the hidden representation from the spatiotemporal backbone, the goal of the Spatial Propagation–Aware Prediction module is to explicitly model the spatial diffusion of pertussis heatmaps based on geographic adjacency and historical transmission trajectories. Let the output feature tensor of the backbone be $\text{F}\in {\mathbb{R}}^{T\times H_{s}\times W_{s}\times C}$, where *T* denotes the number of time steps, *H*_*s*_ and *W*_*s*_ represent the spatial grid partitions in height and width, and *C* is the channel dimension. Its module architecture is shown in Figure 3.

We first flatten the spatial dimensions into node dimensions and apply linear projection to obtain a region-level temporal representation. As shown in Formula 2 and 3


$$F^{\text{flat}}=\text{reshape}(F,T,N,C),\;N=H_{s}W_{s},$$ (2)

$$\begin{array}{l} \text{X}={\text{F}}^{\text{flat}}{\text{W}}_{x}+{\text{b}}_{x},\;{\text{W}}_{x}\in {\mathbb{R}}^{C\times d},\;{\text{b}}_{x}\in {\mathbb{R}}^{d}, \end{array}$$ (3)

where **X**∈ℝ^*T*×*N*×*d*^ represents the *d*-dimensional temporal sequence for each region over the window of length *T*. Based on administrative boundaries or grid-based adjacency, a static adjacency matrix is constructed. As shown in Formula 4


$$\begin{array}{l} \text{A}\in {\mathbb{R}}^{N\times N},a_{ij}=1\;\text{iff regions}i\text{and}j\text{are adjacent; otherwise}a_{ij}=0, \end{array}$$ (4)

which captures the basic spatial topology and provides constraints for subsequent propagation modeling. We acknowledge that infectious disease spread can also be driven by distance-decayed interactions or mobility-mediated connectivity, for which distance-weighted adjacency or mobility-informed matrices are often beneficial. In this study, we adopt administrative adjacency as a parsimonious and reproducible proxy because fine-grained mobility data with consistent temporal coverage are not publicly available in the used surveillance system and may introduce additional privacy and acquisition constraints. Moreover, adjacency-based topology provides a robust minimum-assumption setting to evaluate whether the proposed propagation-aware mechanism can improve diffusion modeling without relying on external mobility priors. Incorporating distance- or mobility-informed connectivity as an additional template/constraint is a natural extension and will be explored when such auxiliary data become accessible.

To characterize propagation intensity under different spatiotemporal patterns, the module employs 1D convolutions along the temporal dimension to extract multiple sets of propagation weights. For region *i*, let ${\text{x}}_{i}\in {\mathbb{R}}^{T\times d}$ denote its temporal feature sequence. Three propagation responses are obtained through three 1D convolution kernels. As shown in Formula 5


$$\begin{array}{l} {\text{z}}_{i}^{\left(k\right)}=\text{Conv}1{\text{D}}_{k}\left({\text{x}}_{i}\right),\;{\text{z}}_{i}^{\left(k\right)}\in {\mathbb{R}}^{m},\;k=1,2,3, \end{array}$$ (5)

where *m* is the output length of each kernel and *k* corresponds to one of three predefined propagation patterns. The temporal responses are then globally averaged into scalar scores. As shown in Formula 6


$$\begin{array}{l} s_{i}^{\left(k\right)}=\frac{1}{m}\overset{m}{\underset{u=1}{\sum }}z_{i,u}^{\left(k\right)},\;k=1,2,3, \end{array}$$ (6)

and normalized via softmax to obtain the weights of region *i* for each propagation pattern. As shown in Formula 7


$$\begin{array}{l} \alpha_{i}^{\left(k\right)}=\frac{\exp\left(s_{i}^{\left(k\right)}\right)}{\sum_{r=1}^{3}\exp\left(s_{i}^{\left(r\right)}\right)},\;k=1,2,3. \end{array}$$ (7)

Given three predefined propagation templates **B**^(*k*)^∈ℝ^*N*×*N*^—representing local adjacency diffusion, temporal inter-region propagation, and self-preserving propagation—the adaptive propagation matrix for the entire graph is constructed by weighted summation. As shown in Formula 8


$$\begin{array}{l} \text{P}=\overset{3}{\underset{k=1}{\sum }}\text{diag}\left(\alpha^{\left(k\right)}\right){\text{B}}^{\left(k\right)}, \end{array}$$ (8)

where **B**^(1)^ = **A** models immediate neighbor spillover, **B**^(2)^ = *I*(**A**^2^>0)−**I**−**A** provides a multi-hop (two-step) proxy of non-local seeding without requiring external mobility data, and **B**^(3)^ = **I** represents within-city persistence. Here, $\alpha^{\left(k\right)}={\left[\alpha_{1}^{\left(k\right)},\ldots ,\alpha_{N}^{\left(k\right)}\right]}^{⊤}$, and diag(**α**^(*k*)^) is a diagonal matrix that modulates region-specific propagation strengths under each mechanism.

With the propagation matrix **P** obtained, a multi-layer graph neural network is used to perform message passing and diffusion modeling. The propagation matrix is symmetrically normalized as shown in Formula 9


$$\begin{array}{l} \tilde{\text{P}}={\text{D}}^{-\frac{1}{2}}\text{P}{\text{D}}^{-\frac{1}{2}},\;{\text{D}}_{ii}=\overset{N}{\underset{j=1}{\sum }}P_{ij}, \end{array}$$ (9)

where **D** is the degree matrix. Using the most recent region features ${\text{X}}_{t}\in {\mathbb{R}}^{N\times d}$ as input, let ${\text{H}}^{\left(l\right)}\in {\mathbb{R}}^{N\times d_{l}}$ denote the node representation at layer *l*. The update rule for layer *l*+1 is: As shown in Formula 10


$$\begin{array}{l} {\text{H}}^{\left(l+1\right)}=\sigma \left(\tilde{\text{P}}{\text{H}}^{\left(l\right)}{\text{W}}^{\left(l\right)}+{\text{b}}^{\left(l\right)}\right),\;l=0,\ldots ,L-1, \end{array}$$ (10)

where ${\text{H}}^{\left(0\right)}={\text{X}}_{t}$, ${\text{W}}^{\left(l\right)}\in {\mathbb{R}}^{d_{l}\times d_{l+1}}$ and ${\text{b}}^{\left(l\right)}\in {\mathbb{R}}^{d_{l+1}}$ are learnable parameters, and σ(·) is an elementwise nonlinearity such as GELU. The output after *L* layers is: As shown in Formula 11


$$\begin{array}{l} \text{Z}={\text{H}}^{\left(L\right)}\in {\mathbb{R}}^{N\times d_{L}}, \end{array}$$ (11)

encoding both propagation paths induced by **P** and multi-hop neighborhood information.

To realign the GNN output back to the grid-based heatmap space, the module first applies linear fusion between node features and original region features to balance propagation-enhanced information with local observations. As shown in Formula 12


$$\begin{array}{l} \text{U}=\lambda \text{Z}+\left(1-\lambda \right){\text{X}}_{t}{\text{W}}_{s},\;{\text{W}}_{s}\in {\mathbb{R}}^{d\times d_{L}}, \end{array}$$ (12)

where λ∈(0, 1) is a learnable scalar parameterized through a sigmoid. The fused features are then reshaped back to the spatial grid and used to generate a propagation-enhanced intensity map. As shown in Formula 13 and 14


$$U^{\text{grid}}=\text{reshape}(U,H_{s},W_{s},d_{L}),$$ (13)

$$\begin{array}{l} {\hat{X}}_{t+1}^{\text{spa}}={\text{W}}_{o}*{\text{U}}^{\text{grid}}+b_{o}, \end{array}$$ (14)

where **W**_*o*_ denotes a 3 × 3 convolution kernel, *b*_*o*_ is a bias term, and ${\hat{X}}_{t+1}^{\text{spa}}\in {\mathbb{R}}^{H_{s}\times W_{s}}$ is the propagation-enhanced predicted heatmap produced by the Spatial Propagation–Aware Prediction module.

### 3.4 Spatial-temporal mixed state output head

**Figure 4:**
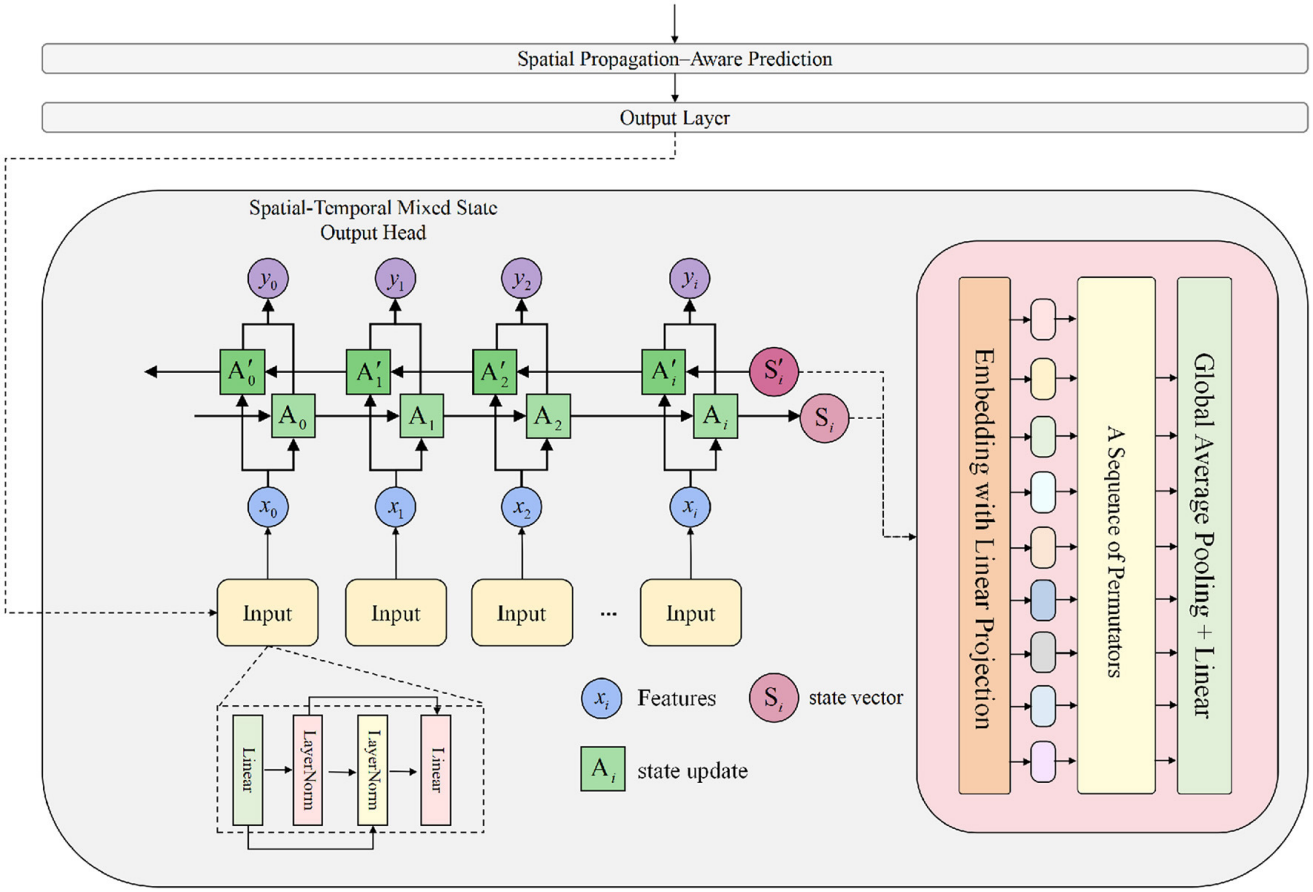
This figure illustrates the structure of the Spatial-Temporal Mixed State Output Head, including linear embedding of input features, position-wise state recursion, state remapping, and a spatiotemporal mixing mechanism based on permutation operations. This module combines linear state space updates with global pooling mapping to jointly decode temporal dependencies and spatial structure information, thereby generating the final heatmap prediction.

The objective of the Spatial–Temporal Mixed State Output Head is to construct an explicit state-recurrence output module that maps temporal dependencies and spatial positional encodings into the next-step heatmap prediction, based on the fused features obtained from the backbone and the Spatial Propagation–Aware Prediction module. The architecture is illustrated in Figure 4.

Let the fused feature tensor be **F**^mix^∈ℝ^*L*×*d*^, where *L* denotes the unfolded spatiotemporal sequence length and *d* is the channel dimension. We first represent the features in sequential form. As shown in Formula 15


$$\begin{array}{l} \text{X}={\left[{\text{x}}_{0},{\text{x}}_{1},\ldots ,{\text{x}}_{L-1}\right]}^{⊤},\;{\text{x}}_{i}\in {\mathbb{R}}^{d}, \end{array}$$ (15)

and map them to the working dimension of the state space via linear embedding. As shown in Formula 16


$$\begin{array}{l} {\tilde{\text{x}}}_{i}={\text{W}}_{e}{\text{x}}_{i}+{\text{b}}_{e},\;{\text{W}}_{e}\in {\mathbb{R}}^{d\times d_{s}},\;{\text{b}}_{e}\in {\mathbb{R}}^{d_{s}}, \end{array}$$ (16)

producing a sequence ${\left\{{\tilde{\text{x}}}_{i}\right\}}_{i=0}^{L-1}$, where *d*_*s*_ denotes the state-space dimension. This ensures that features from the 3DCNN, Mamba backbone, and spatial propagation module enter the state-update process in a unified dimensionality.

To model temporal state evolution, the module adopts a discrete linear state-space recurrence. Let ${\text{s}}_{i}\in {\mathbb{R}}^{d_{s}}$ denote the hidden state at position *i* and ${\text{y}}_{i}\in {\mathbb{R}}^{d_{s}}$ the corresponding intermediate output, with the state initialized as shown in Formula 17


$$\begin{array}{l} {\text{s}}_{0}=\text{0}. \end{array}$$ (17)

State updates are then performed using fixed-dimensional transition and input matrices. As shown in Formula 18 and 19


$$\begin{array}{l} {\text{s}}_{i}=\text{A}{\text{s}}_{i-1}+\text{B}{\tilde{\text{x}}}_{i},\;\text{A},\text{B}\in {\mathbb{R}}^{d_{s}\times d_{s}}, \end{array}$$ (18)

$$\begin{array}{l} {\text{y}}_{i}=\text{C}{\text{s}}_{i}+\text{D}{\tilde{\text{x}}}_{i},\;\text{C},\text{D}\in {\mathbb{R}}^{d_{s}\times d_{s}}, \end{array}$$ (19)

where **A** propagates memory across sequence positions, and **B**, **D** regulate the influence of the current input on the state and output. To enhance the identifiability of output states during downstream aggregation, we introduce a linear state remapping. As shown in Formula 20


$$\begin{array}{l} {\text{s}}_{i}^{\prime }={\text{W}}_{s}{\text{s}}_{i}+{\text{b}}_{s},\;{\text{W}}_{s}\in {\mathbb{R}}^{d_{s}\times d_{s}},\;{\text{b}}_{s}\in {\mathbb{R}}^{d_{s}}, \end{array}$$ (20)

where ${\text{s}}_{i}^{\prime }$ serves as the state feature for subsequent global fusion.

After computing the outputs for all positions, we stack them into matrix form. As shown in Formula 21


$$\begin{array}{l} \text{Y}={\left[{\text{y}}_{0},{\text{y}}_{1},\ldots ,{\text{y}}_{L-1}\right]}^{⊤}\in {\mathbb{R}}^{L\times d_{s}}, \end{array}$$ (21)

and project them to a permutation-compatible embedding space. As shown in Formula 22


$$\begin{array}{l} {\text{Z}}^{\left(0\right)}=\text{Y}{\text{W}}_{p}+{\text{b}}_{p},\;{\text{W}}_{p}\in {\mathbb{R}}^{d_{s}\times d_{p}},\;{\text{b}}_{p}\in {\mathbb{R}}^{d_{p}}, \end{array}$$ (22)

where *d*_*p*_ is the projected feature dimension. To enhance information exchange across spatiotemporal positions, the module applies a sequence of deterministic permutation operators ${\left\{{\text{P}}^{\left(l\right)}\right\}}_{l=1}^{L_{p}}$, where each **P**^(*l*)^∈ℝ^*L*×*L*^ is a permutation matrix with exactly one non-zero entry per row and column. The permutation updates follow: As shown in Formula 23


$$\begin{array}{l} {\text{Z}}^{\left(l\right)}={\text{P}}^{\left(l\right)}{\text{Z}}^{\left(l-1\right)},\;l=1,2,\ldots ,L_{p}, \end{array}$$ (23)

where *L*_*p*_ denotes the number of permutation layers. These repeated reordering operations explicitly mix temporal dependencies and local neighborhood information within the output head.

The final step maps the permuted output back into heatmap space. Let ${\text{z}}_{i}\in {\mathbb{R}}^{d_{p}}$ denote the *i*-th row of ${\text{Z}}^{\left(L_{p}\right)}$. We aggregate the sequence using global average pooling. As shown in Formula 24


$$\begin{array}{l} \bar{\text{z}}=\frac{1}{L}\overset{L-1}{\underset{i=0}{\sum }}{\text{z}}_{i}\in {\mathbb{R}}^{d_{p}}, \end{array}$$ (24)

and obtain the grid-level intensity vector via a linear layer. As shown in Formula 25


$$\begin{array}{l} \text{h}={\text{W}}_{o}\bar{\text{z}}+{\text{b}}_{o},\;{\text{W}}_{o}\in {\mathbb{R}}^{d_{p}\times H_{s}W_{s}},\;{\text{b}}_{o}\in {\mathbb{R}}^{H_{s}W_{s}}, \end{array}$$ (25)

where *H*_*s*_ and *W*_*s*_ denote the grid height and width. Reshaping the output vector yields the predicted heatmap of the Spatial–Temporal Mixed State Output Head. As shown in Formula 26


$${\hat{X}}_{t+1}^{\text{head}}=\text{reshape}(h,H_{s},W_{s}).$$ (26)

Through this combination of state recurrence, permutation-based mixing, and global aggregation, the output head explicitly integrates temporal state information with spatial structural patterns during decoding, providing a structured and interpretable basis for subsequent fusion with the propagation-enhanced branch.

### 3.5 Training objective

To ensure that the proposed spatiotemporal modeling framework effectively learns the fine-grained spatial structure and temporal evolution of pertussis heatmaps, this study adopts the Mean Squared Error (MSE) as the training objective function, measuring the discrepancy between the predicted and ground-truth heatmaps. Since the previously introduced components—3DCNN spatial embedding, Mamba-based state-space modeling, the Spatial Propagation–Aware Prediction module, and the Spatial–Temporal Mixed State Output Head—jointly form a multi-scale feature generation process spanning local structure, temporal dynamics, and propagation pathways, the MSE loss imposes pixel-level consistency constraints on the model output. This encourages the network to remain aligned with true diffusion patterns in terms of spatial distribution, propagation direction, and magnitude of change.

Let the final predicted heatmap be ${\hat{X}}_{t+1}\in {\mathbb{R}}^{H\times W}$ and the corresponding ground truth be $X_{t+1}\in {\mathbb{R}}^{H\times W}$, where *H* and *W* denote the height and width of the spatial grid. The training objective is defined as shown in Formula 27


$$L_{\text{MSE}}=\frac{1}{HW}\sum_{i=1}^{H}\sum_{j=1}^{W}({\hat{X}}_{t+1}(i,j)-X_{t+1}(i,j){)}^{2}.$$ (27)

This loss functions as a global supervisory signal throughout training, guiding the optimization of spatial features, temporal states, and propagation-enhanced representations extracted by the preceding modules under a unified objective. By minimizing the MSE, the model not only learns accurate numerical predictions of regional case intensity but also strengthens spatial coherence and ensures reasonable spatiotemporal evolution in the generated heatmaps, resulting in predictions that more closely resemble real-world diffusion patterns.

## 4 Dataset and experimental setting

### 4.1 Data introduction and data processing

The pertussis spatiotemporal transmission dataset used in this study was obtained from the Infectious Disease Reporting Information Management System of the China Disease Prevention and Control Information System. The filtering criteria included confirmed pertussis cases with a registered residential address in Zhejiang Province and an onset year of 2024. We note that the current study focuses on a single-year cohort primarily because earlier multi-year records with consistent city-level reporting fields and harmonized administrative codes were not accessible to the authors at the time of analysis. Nevertheless, the 2024 daily series still covers a complete annual cycle, providing sufficient within-year temporal span to learn short-term dynamics and seasonal variations within the province. The original case records contained key fields such as onset date and reporting city. To ensure data quality, date formats were standardized, missing or invalid entries were removed, and city names were normalized. All cases were then mapped to a two-dimensional “date–city” space according to their onset dates, producing daily case counts for each prefecture-level city in Zhejiang Province and forming a structured time-series dataset with a “day × city” granularity. At the same time, we acknowledge that limiting the data to 2024 may not fully capture interannual variability driven by changes in immunity, intervention intensity, reporting practices, or broader population mobility patterns; therefore, the generalization of learned patterns across years should be interpreted with caution, and incorporating multi-year data will be an important direction for future validation.

To maintain consistency between case statistics and spatial administrative boundaries, publicly available national administrative-division GeoJSON data were used to extract all prefecture-level city polygons whose administrative codes begin with “33,” corresponding to Zhejiang Province. These boundaries were precisely matched with the cleaned daily case counts to construct a unified and spatially aligned regional dataset. Furthermore, to facilitate deep model learning of spatial transmission patterns, daily case counts for each city were transformed into city-level heatmap representations. This rasterized heatmap format is intended as a modeling representation that preserves spatial adjacency, relative intensity gradients, and cross-city diffusion structure, rather than a replacement for standard epidemiological tabulations. Specifically, under a fixed coordinate system and a unified color range, case counts were filled into their corresponding city polygons, and the maximum annual case count was adopted as the upper limit of the colormap to ensure comparability across dates. We emphasize that the heatmap conversion does not alter the underlying city-level counts; instead, it provides a spatially continuous surface that is convenient for convolutional feature extraction and pattern comparison across time. The generated heatmaps contained no titles, city labels, or color bars in order to emphasize the spatial intensity distribution itself and avoid introducing irrelevant visual cues to the model. Regarding external applicability, this representation can be transferred to other regions by re-projecting local administrative polygons into the same fixed grid and applying consistent normalization; however, differences in boundary granularity, reporting density, or scaling choices may affect interpretability and should be documented when deploying the pipeline beyond Zhejiang.

**Figure 5:**
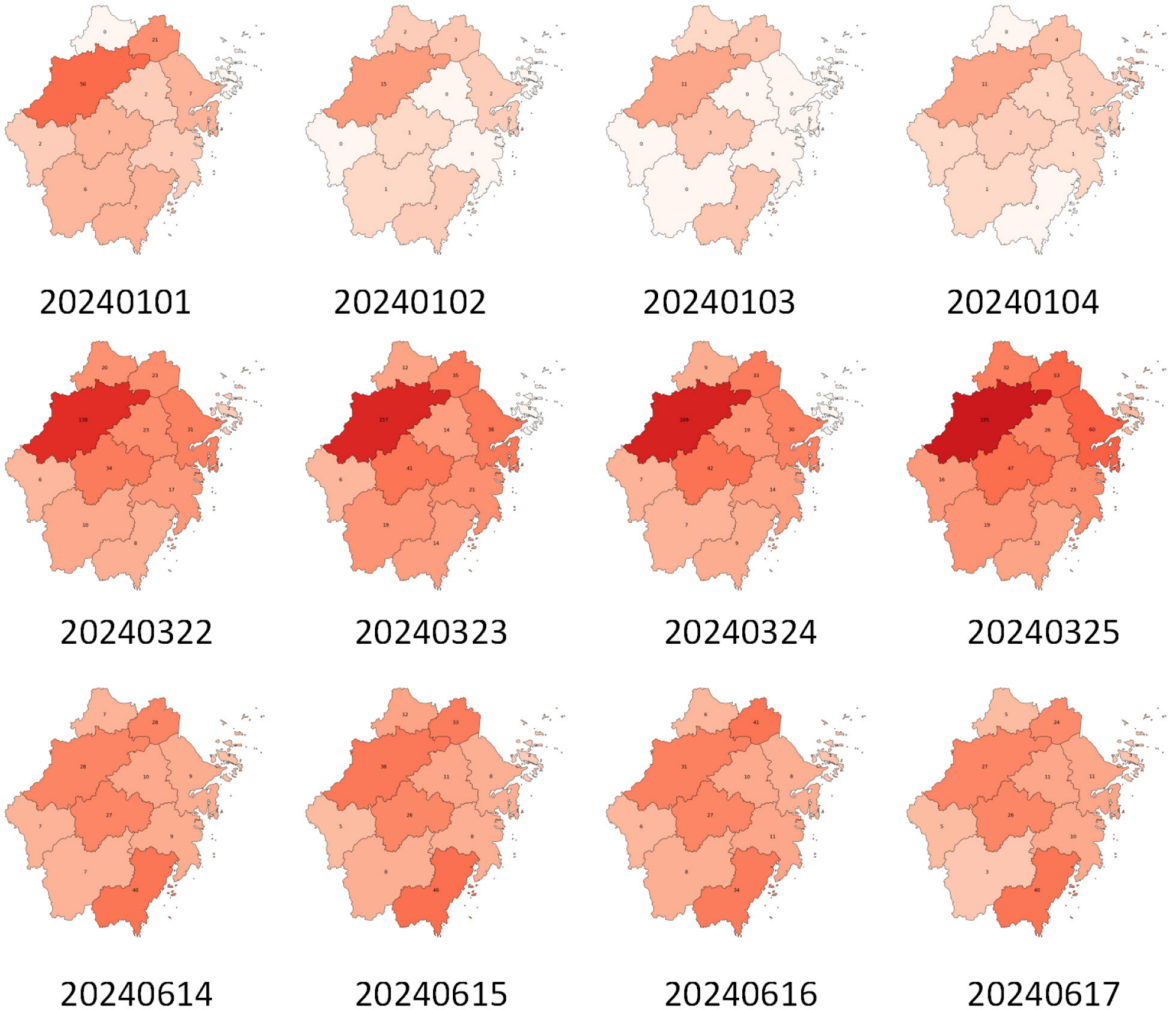
The dataset used in this paper is constructed.

Ultimately, this study constructed a complete sequence of daily pertussis heatmaps for all valid dates in 2024. The data are spatially aligned, temporally continuous, and fine-grained, making them directly suitable as inputs for the subsequent 3D-CNN spatial embedding, Mamba state-space modeling, and spatial propagation prediction modules. An example of the dataset is shown in Figure 5.

### 4.2 Evaluation metric

This study employs four widely used image-level spatiotemporal prediction metrics—Mean Absolute Error (MAE), Mean Squared Error (MSE), Peak Signal-to-Noise Ratio (PSNR), and Structural Similarity Index Measure (SSIM)—to comprehensively assess the quality of pertussis regional heatmap prediction from both numerical error and spatial structural consistency perspectives. MAE evaluates the average absolute deviation between the predicted and ground-truth heatmaps at the pixel level, where a smaller value indicates lower overall prediction bias, and is defined as shown in Formula 28


$$\begin{array}{l} MAE=\frac{1}{HW}\overset{H}{\underset{i=1}{\sum }}\overset{W}{\underset{j=1}{\sum }}|\hat{X}\left(i,j\right)-X\left(i,j\right)|. \end{array}$$ (28)

MSE measures the mean squared prediction error and is more sensitive to large deviations, thereby reflecting the stability of the model in forecasting high-risk regions. Its formulation is given by as shown in Formula 29


$$\text{MSE}=\frac{1}{HW}\sum_{i=1}^{H}\sum_{j=1}^{W}(\hat{X}(i,j)-X(i,j){)}^{2}.$$ (29)

Based on MSE, PSNR converts prediction error into a logarithmic signal-to-noise ratio, quantifying the overall visual fidelity of the prediction relative to the ground truth. It uses the maximum pixel value MAX (set as the global maximum case count in this study) as shown in Formula 30


$$\begin{array}{l} PSNR=10\log_{10}\left(\frac{\text{MA}{\text{X}}^{2}}{\text{MSE}}\right). \end{array}$$ (30)

In addition, SSIM evaluates the structural consistency of the predicted heatmap from the perspectives of luminance, contrast, and structural information, thereby capturing the model's ability to preserve spatial propagation patterns. Let $\mu_{\hat{X}}$ and μ_*X*_ denote the means of the predicted and ground-truth heatmaps, $\sigma_{\hat{X}}^{2}$ and $\sigma_{X}^{2}$ their variances, and $\sigma_{\hat{X}X}$ their covariance. The SSIM metric is formally defined As shown in Formula 31


$$\begin{array}{l} SSIM\left(\hat{X},X\right)=\frac{\left(2\mu_{\hat{X}}\mu_{X}+C_{1}\right)\left(2\sigma_{\hat{X}X}+C_{2}\right)}{\left(\mu_{\hat{X}}^{2}+\mu_{X}^{2}+C_{1}\right)\left(\sigma_{\hat{X}}^{2}+\sigma_{X}^{2}+C_{2}\right)}, \end{array}$$ (31)

where *C*_1_ and *C*_2_ are constants that stabilize the denominator. By jointly considering these four metrics, the evaluation captures error magnitude, sensitivity to extreme deviations, visual consistency, and spatial structural fidelity, thereby providing a rigorous and multidimensional assessment of model performance in pertussis spatiotemporal forecasting. In this study, we adopt pixel-level fidelity and structural-consistency measures because the prediction target is a continuous risk-intensity heatmap rather than discrete outbreak events, and a unified hotspot definition (e.g., thresholding or top-*k* regions) can vary across public-health settings and reporting practices. Nevertheless, we view hotspot-oriented evaluation (e.g., precision/recall of detected high-risk areas under clinically or policy-relevant thresholds) as an important extension and will incorporate such epidemiological metrics when standardized hotspot criteria and externally validated surveillance annotations become available.

## 5 Experimental results

### 5.1 Experimental setting

In terms of the experimental environment, all training and inference procedures of this study were conducted on a server running Ubuntu 22.04. The hardware configuration includes an NVIDIA RTX 3090 GPU with 24 GB of memory, an AMD Ryzen 5950X CPU, and 128 GB of RAM, which together provide sufficient computational capability to support the 3D-CNN spatial convolution, Mamba state-space modeling, and spatial propagation enhancement modules. The software environment is based on Python 3.10, PyTorch 2.2, and CUDA 12.1, complemented by additional libraries such as GeoPandas and Matplotlib for data construction and visualization. All experiments were executed repeatedly under the same configuration to ensure reproducibility and stability of the results.

For hyperparameter settings, the model was trained for 200 epochs using the AdamW optimizer, with an initial learning rate of 1 × 10^−4^, a weight decay of 1 × 10^−4^, and a batch size of 4. The 3D-CNN embedding layer employs 64 channels, while the Mamba state-space backbone consists of 4 layers with a hidden dimension of 128 per layer. The spatial propagation–aware module adopts three parallel 1D-CNN branches, each with 32 channels, followed by a single-layer GNN fusion structure. The output head contains 4 permutation layers. The input window length is set to *T* = 7 days, and the model predicts the next-time heatmap. The main experimental configurations are summarized in Table 1.

**Table 1:** Hyperparameters and runtime environment configuration.

**Category**	**Configuration**
Operating system	Ubuntu 22.04
GPU	NVIDIA RTX 3090 (24GB)
CPU	AMD Ryzen 5950X
Memory	128GB RAM
Deep learning framework	PyTorch 2.2 + CUDA 12.1
Training epochs	200
Batch size	4
Optimizer	AdamW
Learning rate	1 × 10^−4^
Weight decay	1 × 10^−4^
Temporal window length	*T* = 7
3D-CNN channel dimension	64
Mamba layers/hidden size	4 layers / 128 dimensions
Spatial propagation branches	3 × 1D-CNN (32 channels) + 1-layer GNN
Output head permutation layers	4

### 5.2 Experimental results compared with other models

To verify the effectiveness of the proposed spatiotemporal prediction framework, this section systematically compares the model with several existing representative methods, including recurrent networks, convolutional-recurrent hybrid structures, and recent state-of-the-art spatiotemporal modeling models. All methods are evaluated under the same dataset and experimental settings to ensure the fairness and consistency of the comparison results. Through a combination of quantitative metrics and visualization analysis, this section aims to comprehensively demonstrate the overall performance differences of different models in the pertussis heatmap prediction task. The experimental results are shown in Table 2.

**Table 2:** Performance comparison of different methods on the pertussis heatmap prediction task.

**Method**	**MSE**	**MAE**	**PSNR**	**SSIM**
LSTM+Transformer	0.004210	0.04152	27.842	0.9324
CNN+BILSTM	0.003685	0.03841	28.219	0.9417
Bi-PredRNN (44)	0.002931	0.03385	28.904	0.9521
SwinFlood (45)	0.002104	0.02917	29.466	0.9582
PredRNNv3 (46)	0.001782	0.02743	29.812	0.9607
SeeMore (47)	0.001425	0.02489	30.214	0.9650
SwinLSTM (48)	0.001621	0.02533	29.377	0.9611
**Ours**	**0.000930**	**0.020062**	**31.057**	**0.9753**

Bold text represents the best.

From a public health perspective, the central problem addressed in this study is the limited ability of existing spatiotemporal prediction models to provide reliable and fine grained descriptions of infectious disease propagation, especially in regions where transmission exhibits uneven cross-city spread, localized case outbreaks and complex diffusion pathways. Traditional recurrent models and convolution recurrent hybrid frameworks mainly capture local temporal fluctuations, whereas more recent architectures such as SwinFlood and SeeMore improve spatial feature extraction but still have difficulty in jointly modeling geographic adjacency relations and the term evolution of regional transmission patterns. As a result, these approaches tend to produce blurred spatial details or insufficient gradient responses when applied to real public health surveillance scenarios that require accurate identification of changing transmission fronts, shifting risk gradients and emerging hotspots.

In contrast, the proposed method is designed to overcome these practical limitations by integrating three forms of complementary modeling capability that correspond to key determinants of disease diffusion in population level settings. The three dimensional convolutional module represents regional spatial structures and short term dynamic changes, the Mamba state space network captures temporal dependencies that influence the continuity of transmission, and the Spatial Propagation Aware Prediction component explicitly characterizes potential cross-city propagation routes based on administrative adjacency relations. This multi component architecture allows the model to maintain structural consistency and trend accuracy in heatmap prediction. The Spatial Temporal Mixed State Output Head further fuses spatial and temporal information during decoding so that the predicted risk maps more faithfully reflect true diffusion patterns in terms of spatial continuity, boundary clarity and local structural fidelity. As a result, the framework not only reduces numerical prediction errors but also improves interpretability and reliability in depicting essential public health indicators related to propagation dynamics and regional risk distribution. In practical surveillance workflows, these improvements translate into more dependable early warning signals and clearer hotspot delineation, which can support timelier risk stratification and more targeted allocation of monitoring and response resources.

### 5.3 Ablation test results

To further verify the effectiveness of each submodule within the overall framework, this section conducts ablation experiments by constructing multiple progressively evolving model variants. By sequentially adding spatial propagation modeling and spatiotemporal hybrid decoding structures under the same training configuration, the contributions of different components to model expressiveness, spatiotemporal dependency modeling depth, and region propagation consistency can be more clearly observed. Furthermore, ablation experiments help clarify the sources of system performance improvements, thus providing more convincing evidence for the structural rationality and necessity of the model design. The experimental results are shown in Table 3.

**Table 3:** Ablation study results of different model components.

**Method**	**MSE**	**MAE**	**PSNR**	**SSIM**
3DCNN+Mamba	0.001872	0.02851	29.214	0.9542
+SPAP	0.001312	0.02403	30.116	0.9649
+STMSOH	0.001081	0.02218	30.742	0.9706
**Ours**	**0.000930**	**0.020062**	**31.057**	**0.9753**

Bold text represents the best.

The overall trend observed in the ablation study indicates that each module plays a complementary and indispensable role in the construction of the model. The baseline structure composed of 3DCNN and Mamba is responsible for capturing core spatial local structures and temporal dependencies, providing stable and discriminative feature representations for subsequent propagation-aware and decoding mechanisms. However, relying solely on the spatiotemporal backbone is insufficient for modeling the directional characteristics of cross-city case diffusion, which limits the model's ability to capture regional risk gradients and propagation consistency.

With the addition of the Spatial Propagation–Aware Prediction (SPAP) module, the model becomes capable of explicitly modeling potential transmission pathways between adjacent regions, making the learned spatial features more consistent with real-world geographic diffusion processes. When the Spatio–Temporal Mixed State Output Head (STMSOH) is further introduced, the temporal-state fusion and spatial-structure rearrangement mechanisms in the decoding stage enhance the predicted heatmaps in terms of fine-grained details, gradient continuity, and structural alignment. The performance of the full model demonstrates that the three modules form an effective synergy across spatiotemporal representation learning, propagation-structure modeling, and prediction decoding, enabling the framework to more closely reflect the dynamic organizational patterns of pertussis spread and improving both interpretability and reliability of the prediction results.

### 5.4 Ablation statistical analysis

**Figure 6:**
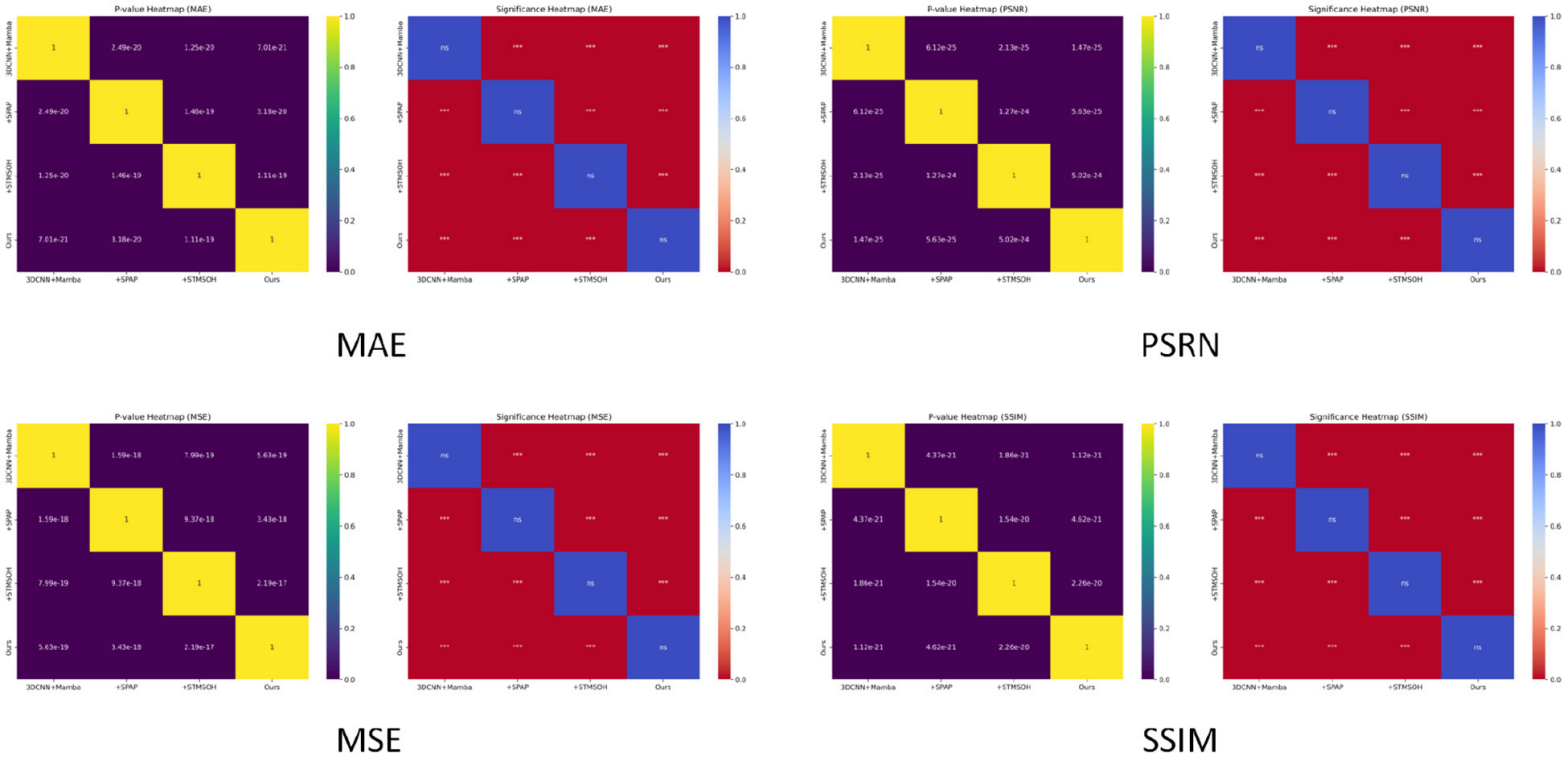
Statistical analysis of experimental results.

To further validate the reliability of the ablation study and ensure that the observed performance variations across different model components are not caused by random fluctuations, we conduct a comprehensive statistical significance analysis. Instead of relying solely on point metrics, this analysis quantitatively evaluates whether the inclusion or removal of each module leads to meaningful performance differences under rigorous statistical testing. Specifically, for each metric we perform pairwise two-sided t-tests on the per-sample evaluation results between any two methods to assess whether their mean performance differs significantly. By incorporating pairwise hypothesis testing and bootstrap-based variance estimation, the study aims to establish the robustness of the architectural design and provide a statistically grounded understanding of how each component contributes to the overall modeling capability. Given the multiple pairwise comparisons involved in the significance matrix, we apply the Benjamini–Hochberg procedure to control the false discovery rate (FDR) across all tested pairs for each metric. The experimental results are shown in Figure 6.

From the significance heatmap of the four evaluation metrics, it can be observed that all diagonal elements are non-significant (ns), whereas all off-diagonal elements exhibit statistically significant differences (typically ***). All significance labels are reported based on the Benjamini–Hochberg adjusted p-values from the two-sided t-tests. This indicates that each method maintains internally consistent performance, while any pair of different methods shows statistically meaningful performance gaps. For both error-based metrics (MSE, MAE) and image-quality metrics (PSNR, SSIM), the introduction of improved modules produces stable and reproducible performance differences across models, ruling out the possibility that the improvements arise from random fluctuations. These results therefore validate the effectiveness of the proposed architectural enhancements.

Furthermore, the structure of the significance matrix shows that each added module contributes substantial improvements in spatiotemporal modeling and predictive reconstruction, with performance gains consistently reflected across multiple dimensions. The final model, as well as the ablated variants, demonstrates significant advantages across all four metrics, suggesting that the designed modules jointly strengthen spatiotemporal feature extraction, propagation-structure modeling, and decoding quality. From a statistical perspective, this provides strong evidence for the superiority and robustness of the complete framework.

### 5.5 Density scatter plot experiment results of predicted and true values

**Figure 7:**
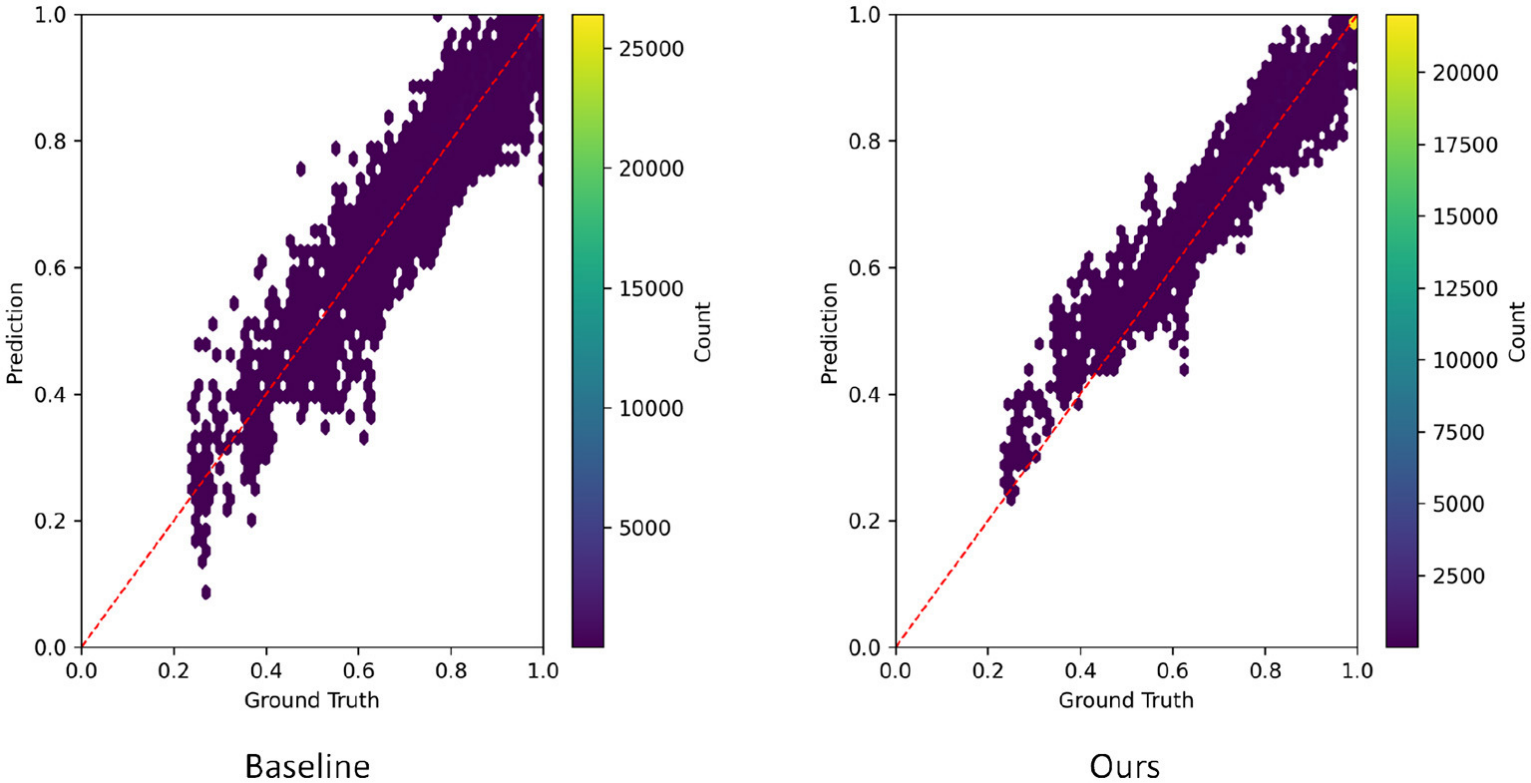
Density scatter plot experiment results of predicted and true values.

This section provides an intuitive assessment of the numerical consistency between the model predictions and the ground-truth values by visualizing a density scatter plot of predicted vs. true values. Compared with relying solely on error metrics, the density scatter plot simultaneously reveals concentration regions, dispersed points, and potential systematic deviations within a two-dimensional space, thereby offering a more comprehensive understanding of the model's fitting behavior and stability across different value ranges. Through this visualization, one can directly identify whether the model exhibits tendencies of systematic underestimation or overestimation and whether the predictions form a high-density cluster around the ideal diagonal line. These observations serve as a fundamental basis for subsequent model diagnosis and performance analysis. The corresponding experimental results are illustrated in Figure 7.

From the density scatter plot, it can be observed that the Baseline model exhibits a wide spread of prediction points around the diagonal, with particularly noticeable dispersion in the medium- and high-risk regions. This scattered distribution indicates systematic bias and high variance in the model's fitting to the true heatmap values. In contrast, the points in the Ours model are much more tightly concentrated near the ideal diagonal line, and the regions of highest density form a slender and continuous structure. This pattern demonstrates that the model maintains more stable and consistent predictions across different risk levels. The transition from dispersed to convergent distributions reflects the effectiveness of the proposed spatiotemporal propagation enhancement and mixed-state modeling mechanisms, which significantly improve alignment with the true diffusion patterns. As a result, the predicted values more accurately capture the global trend, local gradients, and high-intensity regions of the target heatmaps, providing further evidence for the efficiency and robustness of the model design.

### 5.6 Distribution analysis of evaluation metrics for baseline and proposed model

**Figure 8:**
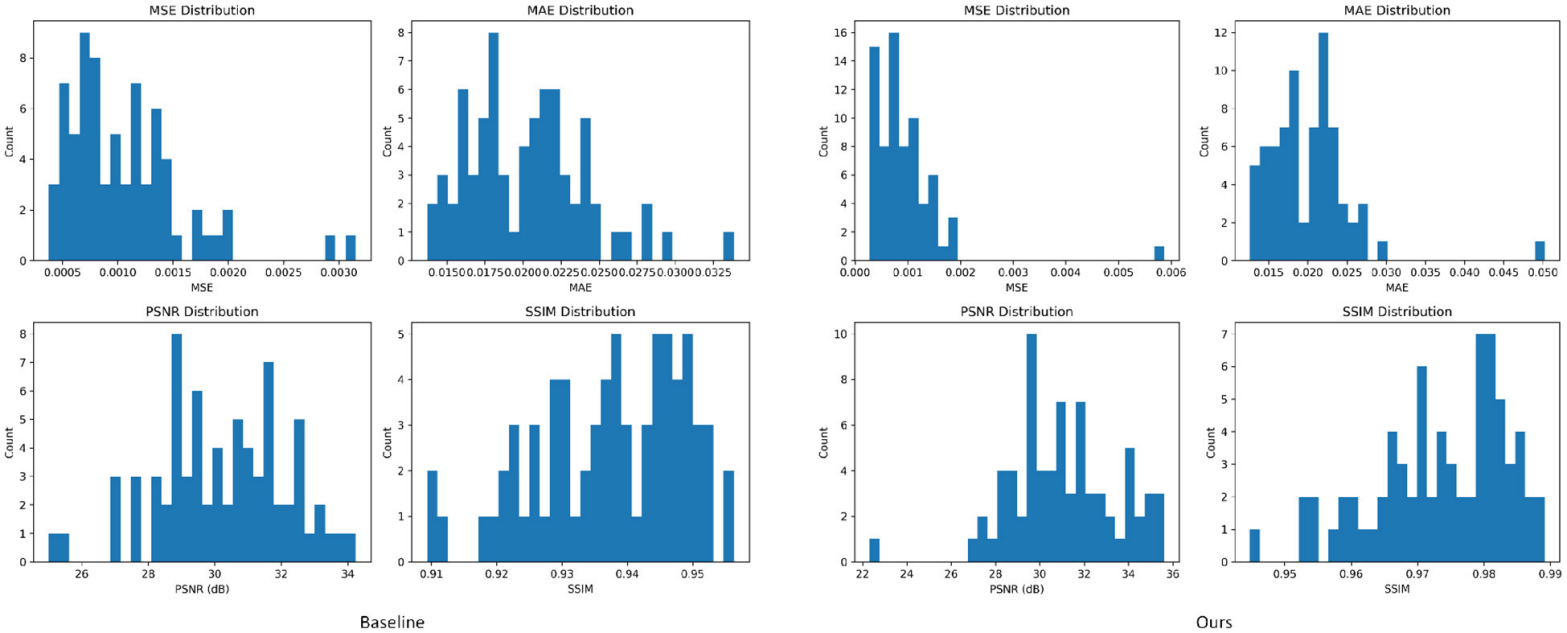
Qualitative visualization of experimental results.

This section conducts a visual analysis of the distribution of various evaluation metrics to further examine the stability of different models in terms of prediction error and structural similarity. By comparing the histogram distributions of MSE, MAE, PSNR, and SSIM for both the Baseline model and the proposed model, it becomes possible to more intuitively observe differences in overall performance variability and error concentration. Such distribution-based visualization provides additional evidence for interpreting the results and understanding the consistency characteristics of each model. The corresponding experimental results are presented in Figure 8.

From the distribution plots of the four evaluation metrics, it can be observed that the error-based indicators (MSE and MAE) of the Baseline model exhibit wider and more dispersed distributions, indicating weaker prediction stability and larger fluctuations across samples. In contrast, the error distributions of the Ours model are noticeably more concentrated with shorter tails, reflecting improved consistency in its overall risk-value regression. Meanwhile, for image-quality and structural-similarity metrics such as PSNR and SSIM, the distributions of the Ours model shift toward higher value ranges and show greater concentration. This demonstrates that the proposed spatiotemporal propagation enhancement and mixed-state output mechanisms enable the model to more stably reproduce the spatial structures and gradient variations of the true heatmaps. These distribution-level observations further validate the accuracy and robustness advantages of the proposed framework.

### 5.7 Qualitative analysis of experimental results

**Figure 9:**
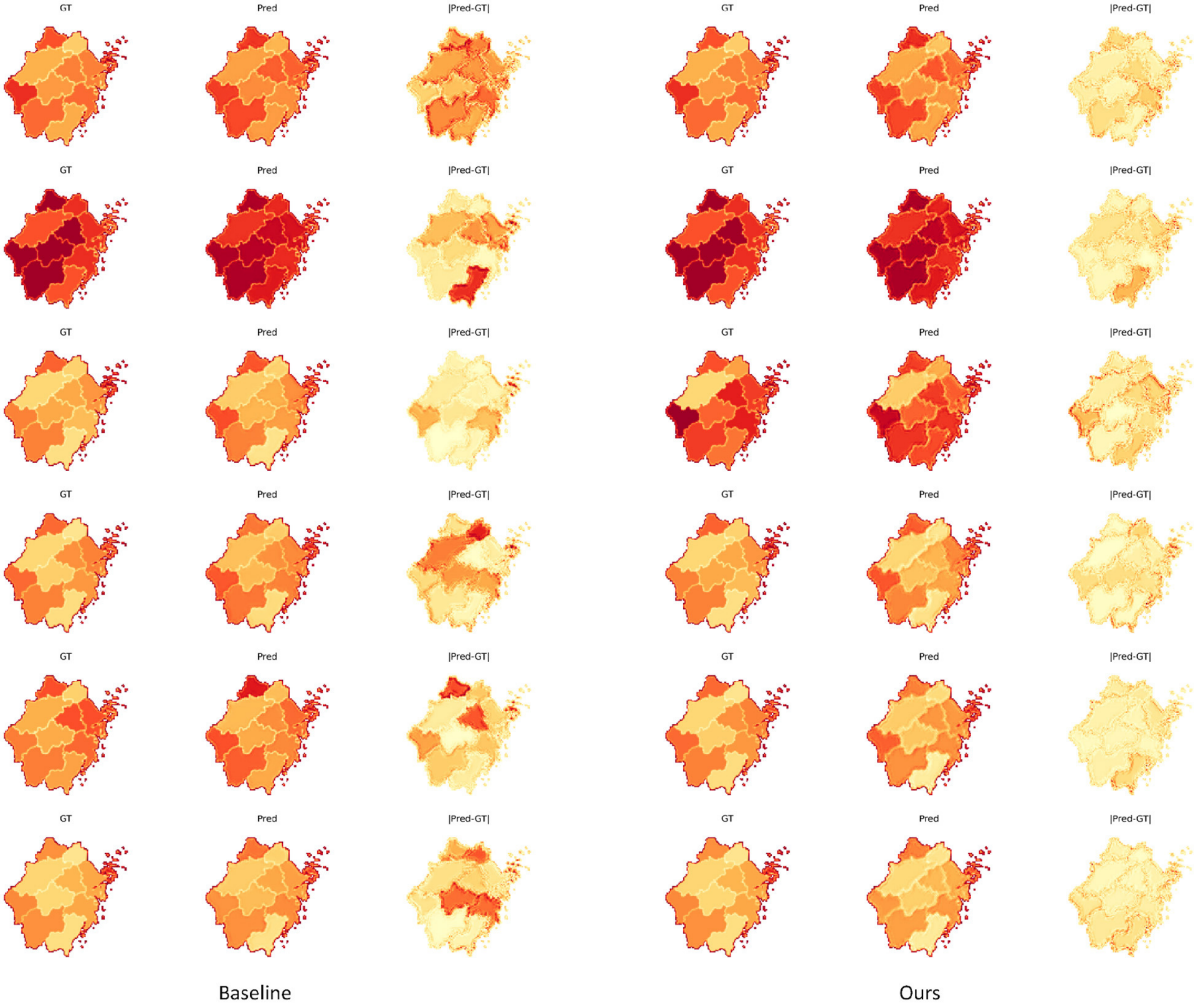
This histogram depicts the stability characteristics of different models in terms of error and structural similarity. These distribution visualizations can help determine the range of fluctuations and central tendency of the models across multiple dimensions from a statistical perspective.

This section provides a qualitative analysis of the model's prediction performance from a visualization perspective, offering a more intuitive demonstration of its capability in spatial structure reconstruction and risk-pattern representation. By comparing the heatmaps generated by different models, one can visually examine the differences in detail recovery and spatial smoothness, thereby offering complementary insights to the quantitative results. The corresponding experimental outcomes are presented in Figure 9.

From the visualization results, it can be observed that the proposed model on the right is able to more completely reconstruct the true spatial diffusion patterns across different time steps. It better preserves the concentration of high-risk regions and produces smoother transitions between neighboring areas, resulting in more faithful heatmap shapes with fewer artifacts. This effectively reduces local fragmentation, boundary blurring, and regional shift effects that appear in the Baseline predictions, where discontinuities and misidentified regions are more frequent. Overall, the proposed model yields more stable visual patterns across time, highlighting the benefit of the spatiotemporal propagation enhancement module and the mixed-state output mechanism for fine-grained alignment and cross-region dynamic modeling.

### 5.8 Data missing limits the analysis of the model

In real-world epidemic monitoring and reporting scenarios, data missingness is often unavoidable, particularly in low-incidence regions or situations with reporting delays. To approximate practical surveillance uncertainty while keeping the evaluation controllable, we simulate missingness by randomly masking a proportion of historical input frames at the pixel level, which corresponds to a Missing Completely at Random stress-test setting; although real pertussis reporting gaps may also exhibit structured non-random patterns (e.g., region-dependent under-reporting or time-lagged submissions), this randomized protocol provides a conservative and reproducible baseline for quantifying robustness under incomplete inputs. To evaluate the model's performance under incomplete input conditions, this section constructs data samples with varying missing rates and analyzes the resulting stability and degradation across key evaluation metrics. The corresponding experimental results are summarized in Table 4.

**Table 4:** Performance under different data missing rates.

**Data missing**	**MSE**	**MAE**	**PSNR**	**SSIM**
15%	0.001150	0.022800	30.20	0.9695
10%	0.001050	0.021700	30.60	0.9720
5%	0.000975	0.020800	30.90	0.9740
0%	0.000930	0.020062	31.057	0.9753

From the results under different missing rates, it can be observed that as the proportion of missing data increases from 5% and 10% to 15%, the model exhibits the expected performance degradation across all evaluation metrics. Specifically, MSE and MAE gradually increase, while PSNR and SSIM consistently decrease. This indicates that incomplete historical inputs weaken the model's ability to capture spatial structures and propagation dynamics, leading to certain deviations in risk-pattern reconstruction. Nevertheless, even under the 15% missing condition, the model maintains relatively high performance, with only moderate fluctuations across the metrics. This demonstrates that the model possesses strong robustness and fault-tolerance when facing real-world monitoring scenarios in which data missingness is likely to occur.

### 5.9 Hyperparameter sensitivity experiment

#### 5.9.1 Input window sensitivity experiment results

This section aims to analyze the impact of different input time window lengths on the model's predictive performance and stability, thereby evaluating the model's sensitivity and robustness to the range of historical information utilization. The experimental results are shown in Table 5.

**Table 5:** Input window sensitivity results under different *T*.

**Setting**	**MSE**	**MAE**	**PSNR**	**SSIM**
*T* = 60	0.002410	0.034852	27.317	0.9482
*T* = 30	0.001820	0.029574	28.557	0.9579
*T* = 14	0.001210	0.023818	30.022	0.9687
*T* = 7	0.000930	0.020062	31.057	0.9753

As shown in Table 5, when the input time window increases from *T* = 7 to *T* = 60, the error metrics (MSE and MAE) consistently increase while the fidelity metrics (PSNR and SSIM) decrease substantially, indicating that overly long historical windows can dilute the effective representation of the current transmission dynamics and introduce additional noise and non-stationary disturbances, which in turn leads to blurred details and degraded structural consistency in the predicted heatmaps. These results suggest that simply extending the range of historical information does not necessarily improve forecasting performance in this surveillance setting; instead, it may reduce the model's ability to focus on salient propagation signals and thus harm overall predictive stability and spatial structure reconstruction quality.

#### 5.9.2 Experimental results of sensitivity to different architectures

This section evaluates the performance fluctuations and overall robustness of the model under structural design changes by comparing experimental results under different network architecture configurations. The experimental results are shown in Table 6.

**Table 6:** Experimental results of sensitivity to different architectures.

**Architecture**	**MSE**	**MAE**	**PSNR**	**SSIM**
BiLSTM	0.001420	0.028531	28.912	0.9581
Transformer	0.001180	0.025374	29.684	0.9647
LSTM	0.001530	0.030245	28.301	0.9524
Mamba	0.000930	0.020062	31.057	0.9753

As shown in Table 6, under the same experimental setting, different sequence modeling architectures lead to notable differences in both prediction error and structural fidelity. Compared with LSTM and BiLSTM, the self-attention-based Transformer achieves overall better performance in terms of MSE/MAE and PSNR/SSIM, suggesting stronger capability in temporal feature integration and spatial structure recovery. Nevertheless, Mamba attains the lowest MSE and MAE while simultaneously yielding the highest PSNR and SSIM, indicating that the resulting latent state representation exhibits higher temporal coherence and discriminative stability under noisy and non-stationary fluctuations. This enables the model to more effectively suppress transient perturbations and emphasize persistent transmission signals, thereby providing a more reliable input representation for downstream spatial propagation enhancement and structured heatmap reconstruction, and ultimately producing finer and more consistent predictions.

## 6 Discussion and limitation

The primary public-health contribution of this study is that it advances case-driven spatiotemporal risk prediction from mere “result presentation” to the generation of actionable risk signals that can support decision-making. By integrating transmission-aware enhancement with structured temporal modeling, we enable early characterization of city-scale pertussis transmission risk and identification of spatial diffusion trends, thereby providing quantitative evidence for health authorities to conduct forward-looking risk stratification, targeted-area surveillance, and intervention prioritization under constrained resources. Compared with conventional analytical frameworks that rely mainly on historical counts or static correlations, our method maintains predictive stability while jointly capturing short-term early-warning cues and cross-regional transmission associations, and outputs pixel-level risk heatmaps that can be readily connected to existing public-health geographic information platforms. This improves front-line interpretability of where risk is emerging, how it may spread, and when it is likely to rise, facilitating rapid situational awareness and timely response.

It should be clearly acknowledged that this work still has several key limitations, and these limitations may affect the generalizability of the conclusions and the effectiveness of real-world deployment. First, the data used in this study cover only a single province (Zhejiang Province) and a single year, meaning that the learned transmission patterns may be influenced by local population mobility structure, healthcare accessibility, and reporting mechanisms; therefore, generalization to inter-provincial or international settings requires cautious evaluation. Second, we indeed lack harmonized historical data from earlier years, which prevents validation of robustness under multi-year contexts such as long-term periodic fluctuations, policy adjustments, or changes in immunity levels, and also limits systematic analysis of inter-annual differences in transmission.

Third, our modeling pipeline is built on routinely reported, region-aggregated surveillance records that are transformed into city-level risk heatmaps, and thus it does not include individual-level clinical and demographic variables such as age, gender, co-morbidities, or severe complications. This constraint may mask heterogeneity across susceptible groups (e.g., infants or immunodeficient patients) and limits direct inference of diagnosis severity, prognosis, or case-fatality risk from the predicted maps. In practice, the proposed framework should be interpreted as a population-level early-warning tool for spatial targeting rather than a patient-level risk stratifier; future extensions can incorporate stratified incidence (e.g., age- or sex-specific aggregates), hospitalization/severity indicators, or harmonized clinical registries to enable subgroup-aware risk mapping and more refined public-health decision support. Meanwhile, for susceptible groups such as infants or immunodeficient patients, emerging non-invasive lung-function assessment tools based on hyperpolarized-gas ventilation MRI may offer complementary evidence for respiratory involvement monitoring and prognosis-oriented follow-up, providing a potential pathway for augmenting subgroup-focused surveillance signals in future deployments (41–43).

For public-health translation, we suggest incorporating this framework as a functional module of early-warning systems or outbreak response tools. For example, it can be linked to routine surveillance data streams at centers for disease control to generate automatically updated daily/weekly risk heatmaps and lists of high-risk cities, which can trigger intensified surveillance, rapid field investigation and resource allocation, targeted catch-up vaccination for key groups, and alert prompts for healthcare facilities. It can also be embedded into emergency command platforms to provide interpretable risk evidence for coordinated regional prevention-and-control actions and for evaluating intervention effects when localized increases or inter-city diffusion signals are detected. Future work will prioritize the incorporation of multi-year and multi-region data and will further examine stability and practical utility through external validation and retrospective back-testing in real operational workflows.

## 7 Conclusions

This study addresses the public health challenge of forecasting regional pertussis risk and proposes a spatiotemporal prediction framework that integrates three dimensional convolutional embeddings, state space modeling and propagation structure enhancement in order to better characterize disease diffusion across cities. The framework is designed around a collaborative mechanism that links local structural modeling, temporal dependency capture and cross-city diffusion characterization so that the generated risk heatmaps can more accurately reflect actual transmission patterns observed in population level surveillance. Systematic comparative experiments, ablation analyses and statistical significance evaluations demonstrate that the proposed method offers substantial improvements in both error based indicators and structural similarity measures. Robustness experiments under different levels of data missingness further confirm that the model maintains stable performance and retains practical applicability for real world public health monitoring tasks.

Although the framework shows strong predictive capability, several public health oriented research directions remain open for further improvement. The use of finer spatial resolution or the incorporation of multi source public health information may enable the model to capture more detailed transmission behaviors at the community level. The introduction of adaptive graph structure learning or the inference of dynamic geographic topology across regions may enhance the generalization ability of the model in settings where spatial relations evolve over time. The integration of the predictive system into public health decision making processes, including the development of risk threshold triggers, early warning strategies and intervention simulation tools, may allow the method to provide more comprehensive support in practical epidemiological applications.

## Data Availability

The original contributions presented in the study are included in the article/supplementary material, further inquiries can be directed to the corresponding author.
